# A geographic distribution database of the Neotropical cassava whitefly complex (Hemiptera, Aleyrodidae) and their associated parasitoids and hyperparasitoids (Hymenoptera)

**DOI:** 10.3897/zookeys.545.6193

**Published:** 2015-12-14

**Authors:** Aymer Andrés Vásquez-Ordóñez, Nicolas A. Hazzi, David Escobar-Prieto, Dario Paz-Jojoa, Soroush Parsa

**Affiliations:** 1 CIAT, Centro Internacional de Agricultura Tropical (CIAT), Apartado Aéreo, 6713 Cali, Colombia; 2 Sección de Entomología, Programa Académico de Biología, Universidad del Valle, Apartado Aéreo, 25360 Cali, Colombia; 3 Departamento de Biología, Universidad de Nariño, Apartado Aéreo, 1175 Pasto, Colombia; 4 UC Davis Chile Life Sciences Innovation Center, Andrés Bello 2299 No. 1102, Providencia, Santiago, Chile

**Keywords:** Aleyrodid, *Manihot esculenta*, hymenopterous parasitoids, hyperparasitism, tritrophic interaction, CIAT’s Arthropod Reference Collection (CIATARC)

## Abstract

Whiteflies (Hemiptera, Aleyrodidae) are represented by more than 1,500 herbivorous species around the world. Some of them are notorious pests of cassava (*Manihot esculenta*), a primary food crop in the tropics. Particularly destructive is a complex of Neotropical cassava whiteflies whose distribution remains restricted to their native range. Despite their importance, neither their distribution, nor that of their associated parasitoids, is well documented. This paper therefore reports observational and specimen-based occurrence records of Neotropical cassava whiteflies and their associated parasitoids and hyperparasitoids. The dataset consists of 1,311 distribution records documented by the International Center for Tropical Agriculture (CIAT) between 1975 and 2012. The specimens are held at CIAT’s Arthropod Reference Collection (CIATARC, Cali, Colombia). Eleven species of whiteflies, 14 species of parasitoids and one species of hyperparasitoids are reported. Approximately 66% of the whitefly records belong to *Aleurotrachelus socialis* and 16% to *Bemisia tuberculata*. The parasitoids with most records are *Encarsia hispida*, *Amitus macgowni* and *Encarsia bellottii* for *Aleurotrachelus socialis*; and *Encarsia sophia* for *Bemisia tuberculata*. The complete dataset is available in Darwin Core Archive format via the Global Biodiversity Information Facility (GBIF).

## Introduction

Whiteflies (Hemiptera, Aleyrodidae) are represented by more than 1,500 herbivorous species around the world (Hodges and Evans 2005, Evans 2007, 2008). Some of them are notorious pests of cassava (*Manihot esculenta*), a primary food crop in the tropics (Lebot 2009). Particularly destructive is a complex of Neotropical cassava whiteflies whose distribution remains restricted to their native range (Trujillo et al. 2004, Bellotti et al 2005). Despite their importance, neither their distribution, nor that of their associated parasitoids, is well documented (Evans 2008, Aliaga 2012, da Silva Alonso et al. 2012, Pietrowski et al 2014, Silva et al. 2014, Plantwise 2015 and Global Biodiversity Information Facility 2015). This paper therefore reports observational and specimen-based occurrence records of Neotropical cassava whiteflies and their associated parasitoids and hyperparasitoids. The dataset consists of 1,311 distribution records documented by the International Center for Tropical Agriculture (CIAT).

## Data published through GBIF

http://www.gbif.org/dataset/c6f4c2de-3b71-4ebd-9c98-c21537548f07

## Project details

**Project title**: Management of RTB Critical Pest and Diseases under Changing Climates, through Risk Assessment, Surveillance and Modeling.

**Project personnel**: Aymer Andrés Vásquez-Ordóñez (Data Manager, Data Publisher), Nicolas A. Hazzi (Data Manager, Data Publisher), Juan David Escobar-Prieto (Data Manager, Data Publisher), Dario Paz-Jojoa (Data Manager, Data Publisher), Rodrigo Zúñiga (Data Manager), Soroush Parsa (Principal Investigator, Data Publisher).

**Whiteflies and parasitoids collectors**: Collectors who have more than 30 records include: Bernardo Arias, Jose A. Castillo, Claudia M. Holguin, José María Guerrero B., Gerardino Perez Francisco Rendon and Harold Trujillo.

**Funding**: This project was supported by the Roots, Tubers and Bananas (RTB) Research Program of the Consultative Group on International Agricultural Research (CGIAR).

**Figure 1.**
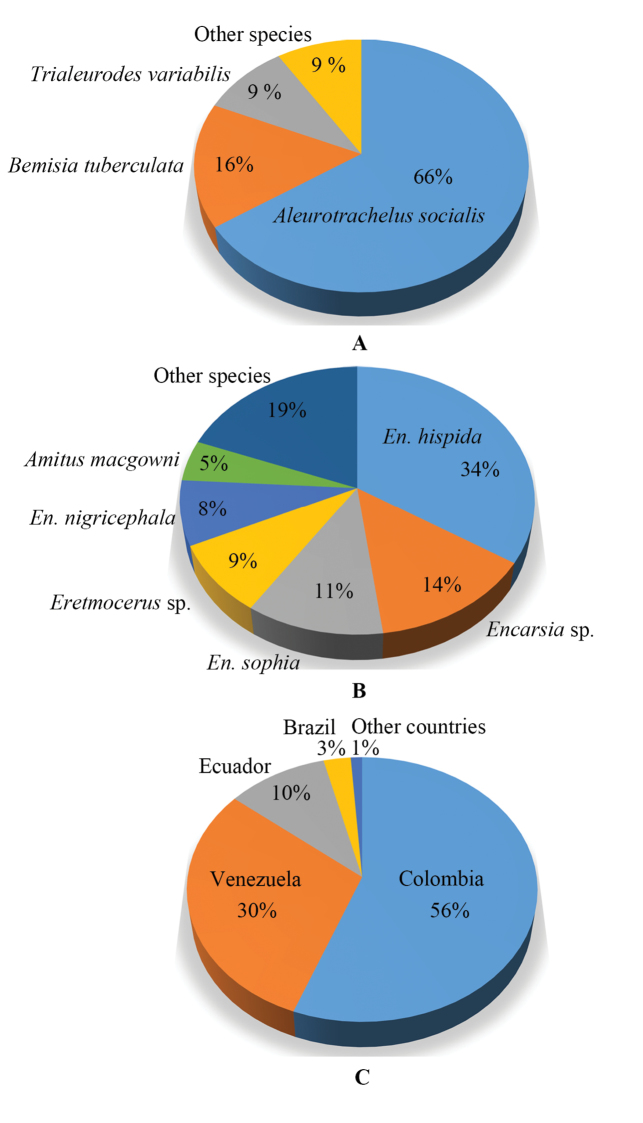
Percentage of occurrence records by Neotropical whitefly species (**A**), by parasitoids (**B**) and by country origin (**C**) in the CIAT’s Arthropod Reference Collection database (N=1,311).

**Design descriptions**: The purpose of this dataset is to broadly and openly share geographic distribution data for the cassava whitefly complex and their associated parasitoids and hyperparasitoids. Prior to this contribution, no records were found of these arthropod species in cassava at the Global Biodiversity Information Facility (2015). To bridge this gap, this paper submits 1,311 distribution records (whiteflies: 841; parasitoids: 466; hyperparasitoids: 4), documented by the International Center for Tropical Agriculture (CIAT) between 1975 and 2012. More than half of these records correspond to specimens preserved at CIAT’s Arthropod Reference Collection (CIATARC). Most of the whitefly records correspond to *Aleurotrachelus socialis* Bondar and *Bemisia tuberculata* Bondar (Fig. 1A). In turn, most parasitoid records belong to *Encarsia hispida* De Santis, *Encarsia* sp. and *Encarsia sophia* (Girault & Dodd) (Fig. 1B). This dataset should be of particular interest to whitefly biologists, cassava entomologists and national plant protection organizations (NPPOs) in tropical countries.

## Taxonomic coverage

### General taxonomic coverage description

Most records were identified to the species level (whiteflies: 97%; parasitoids and hyperparasitoids: 73%) by expert entomologists. Experts identifying more than 20 records were Gregory A. Evans, María del Pilar Hernández, Sueo Nakahara and Louise M. Russell. Whitefly records belong to nine genera and eleven species (Table 1), whereas parasitoid records belong to eight genera and 14 species (Table 1). The dataset also includes four records of the genus *Signiphora* (Table 1), considered a genus of whitefly hyperparasitoids (Evans 2007).

**Table 1.** Neotropical cassava whiteflies or parasitoids associated with the parasitoids and hyperparasitoids of the CIAT’s Arthropod Reference Collection database. Ad: *Aleurodicus dispersus*, Asp: *Aleurodicus* sp., Asu: *Aleuroglandulus subtilis*, Am: *Aleuronudus melzeri*, Aa: *Aleurothrixus aepim*, As: *Aleurotrachelus socialis*, Alsp. *Aleurotrachelus* sp., Bt: *Bemisia tabaci*, Btu: *Bemisia tuberculate*, Bsp: *Bemisia* sp., Tvap: *Trialeurodes vaporariorum*, Tsp: *Tetraleurodes* sp., Tva: *Trialeurodes variabilis*, Trsp: *Trialeurodes* sp., Eh: *Encarsia hispida*, n: number of host for each species.

Hymenoptera		Whitefly species														Eh	n
Family	Species	Ad	Asp	Asu	Am	Aa	As	Alsp	Bt	Btu	Bsp	Tvap	Tsp	Tva	Trsp		
Aphelinidae	*Encarsia* sp.		×	×	×		×		×	×				×	×		8
	*Encarsia americana*						×						×				2
	*Encarsia bellottii*						×							×	×		3
	*Encarsia cubensis*						×										1
	*Encarsia desantisi*			×													1
	*Encarsia guadeloupae*			×													1
	*Encarsia hispida*						×	×	×	×			×	×	×		7
	*Encarsia luteola*								×								1
	*Encarsia nigricephala*								×								1
	*Encarsia pergandiella*									×		×		×			3
	*Encarsia sophia*						×		×	×	×						4
	*Encarsia tabacivora*								×						×		2
	*Eretmocerus* sp.						×		×	×				×			4
Ceraphronidae	*Aphanogmus* sp.						×										1
Encyrtidae	*Anagyrus* sp.					×											1
	*Metaphycus* sp.						×			×							2
Eulophidae	*Aleuroctonus vittatus*	×															1
	*Euderomphale* sp.						×			×							2
Platygastridae	*Amitus* sp.						×			×				×			3
	*Amitus fuscipennis*												×				1
	*Amitus macgowni*		×				×	×									3
Signiphoridae	*Signiphora* sp.						×^1^									×	2
	*Signiphora aleyrodis*									×^1^							1
**Total species by host**		1	2	3	1	1	13	2	7	9	1	1	3	6	4	1	

1 This is a hyperparasitoid case (see taxonomic coverage).

## Taxonomic ranks

**Kingdom**: Animalia

**Phylum**: Arthropoda

**Class**: Insecta

**Order**: Hemiptera, Hymenoptera

**Family**: Aleyrodidae, Aphelinidae, Ceraphronidae, Encyrtidae, Eulophidae, Platygastridae, Signiphoridae

**Genus**: *Aleuroctonus*, *Aleurodicus*, *Aleuroglandulus*, *Aleuronudus*, *Aleurothrixus*, *Aleurotrachelus*, *Amitus*, *Anagyrus*, *Aphanogmus*, *Bemisia*, *Encarsia*, *Eretmocerus*, *Euderomphale*, *Metaphycus*, *Paraleyrodes*, *Signiphora*, *Tetraleurodes*, *Trialeurodes*

**Species**: *Aleuroctonus vittatus* (Dozier), *Aleurodicus dispersus* Russell, *Aleurodicus flavus* Hempel, *Aleuroglandulus subtilis* Bondar, *Aleurothrixus aepim* (Goldi), *Aleurotrachelus socialis* Bondar, *Amitus fuscipennis* MacGown & Nebeker, *Amitus macgowni* Evans & Castillo, *Bemisia tabaci* (Gennadius), *Bemisia tuberculata* Bondar, *Encarsia americana* (DeBach & Rose), *Encarsia bellotti* Evans & Castillo, *Encarsia cubensis* Gahan, *Encarsia desantisis* Viggiani, *Encarsia guadeloupae* Viggiani, *Encarsia hispida* De Santis, *Encarsia luteola* Howard, *Encarsia nigricephala* Dozier, *Encarsia pergandiella* Howard, *Encarsia sophia* (Girault & Dodd), *Encarsia tabacivora* Viggiani, *Signiphora aleyrodis* Ashmead, *Tetraleurodes ursorum* (Cockerell), *Trialeurodes similis* Russell, *Trialeurodes vaporariorum* (Westwood), *Trialeurodes variabilis* (Quaintance)

**Common name**: whitefly (for Aleyrodidae)

## Spatial coverage

**Figure 2.**
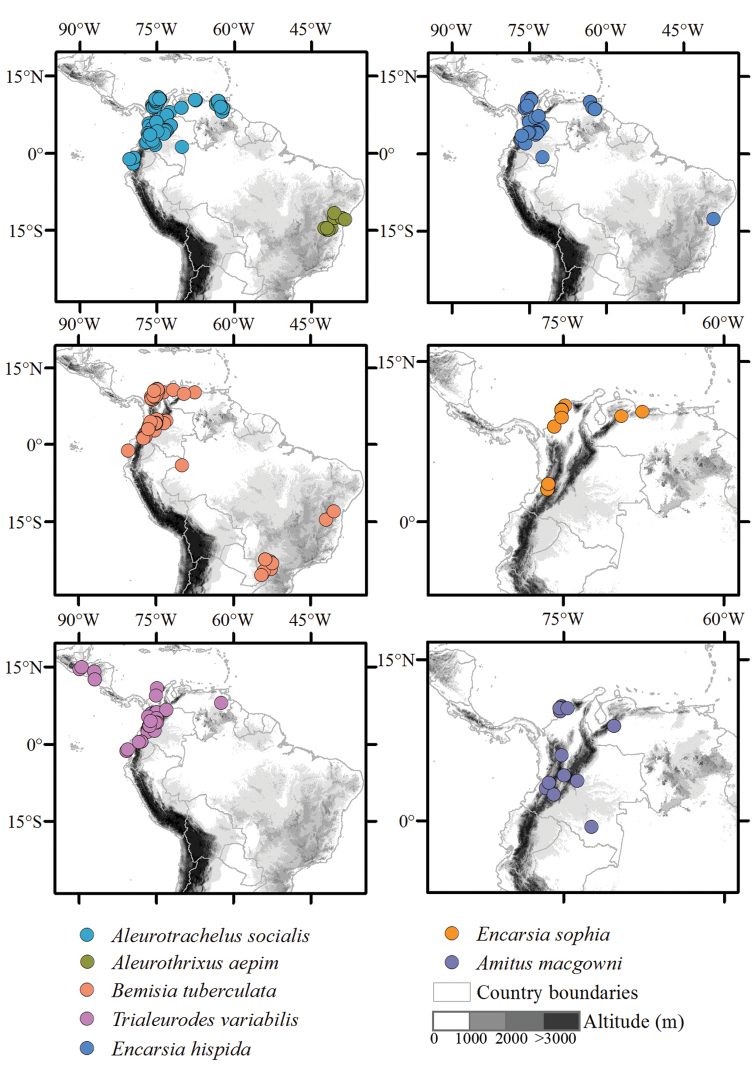
Geographic distributions of Neotropical cassava whitefly species (maps on the left) and their associated parasitoid species (maps on the right) in the CIAT’s Arthropod Reference Collection database.

**General spatial coverage**: Most of the distribution records belong to South America (Brazil, Colombia, Ecuador and Venezuela) and Central America (El Salvador, Guatemala, Honduras, Nicaragua and Panama). Colombia and Venezuela are the best represented countries, followed by Brazil and Ecuador (Fig. 1C). There are also seven records of whiteflies from Asia (Lao and Thailand). The distribution maps of principal whiteflies and their parasitoids are shown in Figure 2.

**Coordinates**: 17.95751 and -25.38936 latitude; -89.86917 and 104.72175 longitude

**Temporal coverage**: 1975-2012

## Natural collections descriptions

**Collection name**: CIAT’s Arthropod Reference Collection (CIATARC)

**Specimen preservation method**: Specimens are preserved in microslides (whiteflies, parasitoids and hyperparasitoids), tissue beds on dried vials (parasitoids), 70% ethyl alcohol (parasitoids and hyperparasitoids), or in 35 mm plastic slide mounts (whiteflies). These samples are deposited within cabinet drawers maintained at 21.0 ± 0.4 °C and 47.6 ± 8.6% relative humidity. They are sorted numerically by species and country of origin.

**Curatorial unit**: 1601 with an uncertainty of 0.

## Methods

**Method step description**: The dataset integrates two data flows: observational records and specimen-based records, identified either to genus or to species. The former were digitized from field diagnostic forms recorded by personnel extensively trained in identification of whiteflies and parasitoids identification. These identifications, however, were likely conducted on site without mounting and preserving samples. Alternatively, these observations may correspond to properly-mounted but lost specimens. In either case, we are significantly confident on these identifications due to relatively clear macroscopic differences in our focal taxa (Caballero 1994, Fernández and Sharkey 2006). Still, conservative users of our database may prefer to rely only on genus-level identifications of these records. On the other hand, the specimen-based records belong to verifiable samples properly-preserved at CIATARC. Guidelines of Martin (1987) and Hodges and Evans (2005) were followed for whitefly slide preparations, and Noyes (1982) for parasitoid and hyperparasitoid preparations. Unique accession numbers were assigned to all records.

All biodiversity data available (i.e. specimen, species identification, name of determiner, sex, locality, date, habitat, host, collector and observations) were digitized in a Microsoft Excel 2010 spreadsheet adopting the Darwin Core Archive format v1.2 (Wieczorek et al. 2012). We updated locality fields (e.g., district, municipality) using the most current names and classifications of administrative divisions used by each country (e.g. http://www.dane.gov.co/Divipola/ for Colombia, http://www.inec.gob.ec/estadisticas/?option=com_content&view=article&id=80 for Ecuador, etc. [accessed 14 November 2014]). Based on their locality names, we then geocoded the records using Google Maps (https://maps.google.com/), Geolocate (http://www.museum.tulane.edu/geolocate/), GeoNames (http://www.geonames.org/) or with georeference indicated in scientific articles (Calderón et al. 1994, Eiszner et al. 1996, Navia Estrada et al. 2006, Cuadros et al. 2011, Gutiérrez R. et al. 2011). GPS coordinates were converted to decimal degrees. The dataset with metadata was uploaded to the Integrated Publishing Toolkit (IPT) of the Colombia node of Global Biodiversity Information Facility (GBIF) (http://www.gbif.org/dataset/c6f4c2de-3b71-4ebd-9c98-c21537548f07).

**Sampling description**: The records in the dataset have been documented in three ways:

1) Records from CIAT’s initial field explorations to document pests in cassava (CIAT 1974, 1985; 0.7% records, between 1975-1989).

2) Records documented during the “Biological Control of Whiteflies by Indigenous Natural Enemies for Major Food Crops in the Neotropics Projects” and participation in “Global Whitefly IPM Project” led by CIAT, Instituto Nacional de Investigaciones Agropecuarias (INIAP), Centro Nacional de Investigaciones Agropecuarias (CENIAP), Empresa Brasileira de Pesquisa Agropecuária (EMBRAPA), The University of Florida and Corporación Colombiana de Investigacion Agropecuaria (Corpoica) (CIAT 1995, 2002, Bellotti et al. 1996, 1999, 2000, 2005, Bellotti 2001, Arias and Bellotti 2002, CIAT et al. 1998, Castillo 1996, López-Ávila et al. 2001, Hernandez and Bellotti 2002, Holguín et al. 2002, Hernández et al. 2004, 2009, Trujillo et al. 2004, Herrera et al. 2006; 95.7% records, between 1990-2007).

3 Records from other sources; including field inspections and collections conducted during routine farm visits by CIAT personnel, and specimens submitted to CIATARC by fellow institutions and researchers (Adriano Muñoz and Gerardino Perez, pers. comm. November 29, 2014; 2.6% records between 2008-2012).

The records resulted from one of two sampling methods. The first method was designed to identify parasitoids associated with dominant whitefly species on farmers’ fields. One middle-canopy leaf infested with whiteflies was collected from each of 40-100 randomly-selected plants per field. A disc of 2.54 cm^2^ was excised from the leaf lobe with the highest density of whitefly pupae. The single most abundant whitefly species per disc was identified and individuals not belonging to that species were eliminated by puncturing them with a needle. The disc samples were then individually placed in 25-ml glass vials and held for 2–3 days at 24.5 ± 4 °C and 70 ± 5% relative humidity under laboratory conditions until parasitoids emerged (Bellotti et al. 1999, 2000, Trujillo et al. 2004). The second method corresponds to opportunistic collections during routine farm visits by CIAT personnel, when leaves infested with whitefly pupae would be collected in vials with 70% alcohol and submitted to the CIATARC for subsequent identification (Herrera et al. 2006). All formally-submitted samples were mounted and are preserved at the CIATARC. The database does not indicate which sampling method was used for each record.

**Quality control description**: Record validation and cleaning was incorporated at several steps of the documentation process, following guideless by Chapman (2005a, b). The scientific names on labels were checked with a taxonomic thesaurus developed by Aymer Andrés Vásquez-Ordóñez, Juan David Escobar-Prieto and Dario Paz-Jojoa. This thesaurus compiled all known synonyms and spelling variants of the scientific names used for our focal species. Scientific names were assigned in accordance to current taxonomic trends (whiteflies: Evans 2008; parasitoids and hyperparasitoids: Woolley 1988, Polaszek et al. 2004, Evans 2007, Johnson 2007, 2015, Noyes 2014; associated plants: Tropicos 2014). Geographic coordinates were verified using the “Check Coordinates” function in DIVA-GIS (Hitmans et al. 2001). For this last step, we relied on the Global Administrative Unit Layers (GAUL) shape file developed by the Food and Agriculture Organization of the United Nations (FAO 2015), and official shape of administrative division of Brazil, Colombia, Ecuador and Venezuela (IBGE 2007, INEC 2011, SIGOT 2011, IVIC 2007).

## Datasets

### Dataset description

**Object name**: Darwin Core Archive cassava whiteflies complex and their associated parasitoids and hyperparasitoids: data of the CIAT’s Arthropod Reference Collection of International Center for Tropical Agriculture (CIAT).

**Character encoding**: UTF-8

**Format name**: Darwin Core Archive format

**Format version**: 1.0

**Distribution**: http://www.gbif.org/dataset/c6f4c2de-3b71-4ebd-9c98-c21537548f07

**Publication date of data**: 2015-05-15

**Language**: English

**Licenses of use**: This dataset [Neotropical cassava whiteflies complex and their associated parasitoids and hyperparasitoids of CIAT's Arthropod Reference Collection (CIATARC)] is made available under the Creative Commons Zero (CC0) 1.0.
